# Use of high-density tiling microarrays to identify mutations globally and elucidate mechanisms of drug resistance in *Plasmodium falciparum*

**DOI:** 10.1186/gb-2009-10-2-r21

**Published:** 2009-02-13

**Authors:** Neekesh V Dharia, Amar Bir Singh Sidhu, María Belén Cassera, Scott J Westenberger, Selina ER Bopp, Rich T Eastman, David Plouffe, Serge Batalov, Daniel J Park, Sarah K Volkman, Dyann F Wirth, Yingyao Zhou, David A Fidock, Elizabeth A Winzeler

**Affiliations:** 1 Department of Cell Biology, ICND 202, The Scripps Research Institute, North Torrey Pines Road, La Jolla, CA 92037, USA; 2 Department of Microbiology, Columbia University College of Physicians and Surgeons, West 186th Street, New York, NY 10032, USA; 3 The Broad Institute of MIT and Harvard, Cambridge Center, Cambridge, MA 02142, USA; 4 Department of Biochemistry, Albert Einstein College of Medicine at Yeshiva University, Morris Park Avenue, Bronx, NY 10461, USA; 5 Genomics Institute of the Novartis Research Foundation, John Jay Hopkins Drive, San Diego, CA 92121, USA; 6 The Broad Institute of MIT and Harvard, Cambridge Center, Cambridge, MA 02142, USA; 7 Department of Immunology and Infectious Diseases, Harvard School of Public Health, Huntington Avenue, Boston, MA 02115, USA; 8 School for Health Studies, Simmons College, The Fenway, Boston, MA 02115, USA; 9 Department of Medicine, Columbia University College of Physicians and Surgeons, West 186th Street, New York, NY 10032, USA

## Abstract

**Background:**

The identification of genetic changes that confer drug resistance or other phenotypic changes in pathogens can help optimize treatment strategies, support the development of new therapeutic agents, and provide information about the likely function of genes. Elucidating mechanisms of phenotypic drug resistance can also assist in identifying the mode of action of uncharacterized but potent antimalarial compounds identified in high-throughput chemical screening campaigns against *Plasmodium falciparum*.

**Results:**

Here we show that tiling microarrays can detect *de novo *a large proportion of the genetic changes that differentiate one genome from another. We show that we detect most single nucleotide polymorphisms or small insertion deletion events and all known copy number variations that distinguish three laboratory isolates using readily accessible methods. We used the approach to discover mutations that occur during the selection process after transfection. We also elucidated a mechanism by which parasites acquire resistance to the antimalarial fosmidomycin, which targets the parasite isoprenoid synthesis pathway. Our microarray-based approach allowed us to attribute *in vitro *derived fosmidomycin resistance to a copy number variation event in the *pfdxr *gene, which enables the parasite to overcome fosmidomycin-mediated inhibition of isoprenoid biosynthesis.

**Conclusions:**

We show that newly emerged single nucleotide polymorphisms can readily be detected and that malaria parasites can rapidly acquire gene amplifications in response to *in vitro *drug pressure. The ability to define comprehensively genetic variability in *P. falciparum *with a single overnight hybridization creates new opportunities to study parasite evolution and improve the treatment and control of malaria.

## Abstract 2

Using tiling microarrays, a mechanism by which Plasmodium falciparum parasites acquire resistance to the antimalarial fosmidomycin has been elucidated

## Background

With many complete eukaryotic genomes and draft eukaryotic sequencing projects deposited in the National Center for Biotechnology Information database, attention is shifting to discovering genomic diversity and associating this genetic variation with defined phenotypes. This is of particular interest with the human malarial parasite *Plasmodium falciparum*, whose extensive genetic variability and sexual recombination facilitates the emergence and spread of drug resistance [1,2], resulting in treatment failure for many of the licensed antimalarial agents [3,4]. Identifying the genetic changes that are involved in drug resistance or other phenotypic changes can help with the development of effective therapies, improve understanding of parasite biology and gene function, and assist in elucidating the mode of action of uncharacterized chemical compounds that exhibit antimalarial activity in high-throughput cellular screening campaigns [5-7]. Traditional genetic methods have been used to discover such genetic changes but with much difficulty, time, and cost for the experimentally intractable *P. falciparum*.

Traditional forward genetic methods that have been used to discover *Plasmodium *genes involved in drug resistance include genetic crosses and analysis of linkage patterns of sexual assortment that occur naturally during parasite transmission from mammal to insect. For example, the primary genetic determinant of chloroquine drug resistance in *P. falciparum *was identified through a costly genetic cross involving chimpanzees [8,9]. Allelic replacement experiments confirmed that resistance was mediated by point mutations in the chloroquine resistance transporter (*pfcrt*, MAL7P1.27) [10]. Crosses can also be performed at a significantly reduced cost using rodent malaria models, but the mechanism of drug resistance in these systems may not extend to human malaria [11]. In certain instances, linkage disequilibrium studies of sensitive and resistant field isolates, using single nucleotide polymorphisms (SNPs) reported by the recent sequencing projects [12-14], can also uncover genetic determinants of resistance. Indeed, recent analysis of such data has identified selective sweeps associated with chloroquine and antifolate drug resistance [12,15].

An alternative reverse genetic approach leverages knowledge from other systems to predict the candidate genes that might be involved in antimalarial drug resistance. For instance, membrane transporters encoded by multidrug resistance (*mdr*) genes can contribute to drug resistance in other organisms. In the case of *P. falciparum*, amplification of the *pfmdr1 *gene (PFE1150w) leads to mefloquine resistance [16,17], and point mutations in this gene modulate *in vitro *susceptibility to multiple antimalarial agents [1,18,19]. SNPs in the dihydrofolate reductase-thymidylate synthase gene (*pfdhfr-ts*, PFD0830w) confer resistance to antifolate drugs [1,18,20], and a candidate gene approach has been used to successfully correlate *in vitro *derived resistance to the macrolide azithromycin with a point mutation in a ribosomal protein that is part of the apicoplast translation machinery [21]. These candidate gene approaches, however, have limited predictive value with drugs that are specific to malarial parasites and have unknown modes of action.

Not withstanding some earlier successes with classical genetic approaches, technological advances in genomics research are beginning to afford unprecedented power in genome-wide discovery of mutations, thus facilitating high-throughput approaches for discovering genes that are involved in drug resistance. Particularly in laboratory-adapted isogenic isolates, genomic methods are making it possible to differentiate between genetic changes associated with resistance and random mutations. Genome re-sequencing has proven especially useful for SNP detection; however, its primary limitation is in not being able to detect copy number variations (CNVs), which are likely to be a common response to drug pressure. Alternative microarray-based approaches have been used to discover a novel amplification event surrounding GTP cyclohydrolase I (*pfgch1*, PFL1155w) that may be important for antifolate drug resistance [22,23]. Microarray approaches can rapidly identify variable genomic regions and assess CNVs at relatively low cost, providing a distinct advantage over conventional sequencing approaches.

Here we describe the production of a custom high-density tiling 25mer oligonucleotide microarray, based on the 3D7 reference isolate, and the development of analytical tools for the genome-wide identification of mutations in laboratory and clinical isolates of *P. falciparum*. This enabled us to identify more than 90% of reported SNPs in Dd2 with respect to 3D7, and the precise mapping of amplification events surrounding *pfmdr1 *and *pfgch1*, all in a fast, single hybridization experiment. We illustrate the utility of this approach by reporting evidence of a molecular and biochemical basis of parasite resistance to fosmidomycin: amplification of the gene encoding the putative target *P. falciparum *1-deoxy-D-xylulose 5-phosphate reductoisomerase (*pfdxr*, PF14_0641) in parasite lines selected for decreased fosmidomycin susceptibility *in vitro*.

## Results

### Microarray design

One goal of this study was to determine whether we could use hybridization methods to detect SNPs and CNVs globally in the *P. falciparum *genome and to implement a robust and user-friendly software package to analyze these data. We first constructed a custom high-density microarray containing over 4.8 million probes at 5 μm feature size to the sequenced 3D7 isolate [24]. The microarray covers approximately 90% of coding regions, which comprise 53% of the genome, and 60% of noncoding regions, and it is only limited by the high AT content of the *P. falciparum *genome [24]. Unlike the yeast tiling microarray [25], our microarray contains only perfect match probes, and thus we were able to tile through the genome with overlapping probes of alternating strandedness averaging 25 nucleotides in length with a base pair spacing of two to three nucleotides. Thus, each nucleotide in the coding genome could be probed from 9 to 13 times on the microarray. This probe density allows for detection of polymorphisms within a distance of several nucleotides, and the density is much greater than our previous *P. falciparum *microarray that had 327,989 non-overlapping 25 mer oligonucleotide probes spaced approximately every 50 base pairs [22] or the microarray, also used for SNP and CNV detection, designed at The Sanger Institute that has about 2.5 million probes [26].

To validate our microarray-based approach, genomic DNA preparations from the culture-adapted Dd2, HB3, and 3D7 parasite lines were labeled and hybridized to the microarray. We used these lines as references because of the availability of high-quality sequence data generated by traditional sequencing methods. Following hybridization, the arrays were washed and scanned, and the microarray data normalized to a baseline synthetic array using the nonlinear piece-wise running median line for invariant probes [27]. This synthetic array was constructed by taking the mean for each probe across all arrays for analysis. This method of normalization was chosen over quantile normalization [27], a popular method for gene expression analysis, because the reference isolate and other isolates were expected to have different distributions of probe intensities as a consequence of their natural genetic variation.

### Gene amplification and deletion events

We next sought to determine whether we could detect known CNVs and deletions using our microarray-based approach, in view of the key role that CNVs can play in the phenotypes of diverse organisms, including malarial parasites. For this analysis, we used only probes that mapped to only one location in the 3D7 reference genome (87.7% of total probes). The data were subject to two different analyses. First, we systematically detected gene deletion events using a match-only integral distribution (MOID) algorithm [28], similar to one previously used in our laboratory to detect CNVs with the previous generation of microarrays [22]. For this analysis, we modeled the background hybridization by using Affymetrix eukaryotic background control probes. This required at least ten unique probes per gene, and thus we excluded 95 highly variable subtelomeric genes (Additional data file 1). We considered one of two criteria to designate a gene as absent: the probe hybridization results fell within the distribution of background probes with a *P *value cutoff of 1 × 10^-2^; or the gene had less than a 2.5-fold increase in intensity at the 70th percentile with respect to background. Using the MOID algorithm, we identified a total of 5,461 genes with ten or more probes in 3D7 as present, indicating a low false-positive rate. We also confirmed that a group of six subtelomeric cytoadherence genes on chromosome 2 were deleted in Dd2 compared with 3D7, in accordance with the findings of a previous report [22] (Additional data file 1).

**Figure 1:**
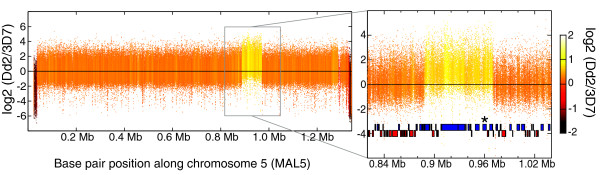
Amplification event surrounding *pfmdr1 *on chromosome 5 in Dd2. The log_2 _ratio of the intensity of each unique probe in Dd2 was divided by the intensity of each unique probe in 3D7 to generate the plot. The probe log ratios were colored by the moving average over a 500 base pair window as indicated in the color bar. A normal distribution around zero was expected if the copy number was the same. The ends of chromosome 5 are highly polymorphic subtelomeric regions and thus showed much lower probe intensity in Dd2 when compared with the reference 3D7. The amplification containing 14 genes including *pfmdr1 *(marked with an asterisk) is enlarged in the inset. Mb, megabases.

Second, we detected amplification events by determining the log_2 _ratio of unique probes in the isolate compared with the reference isolate. Each chromosome was scanned with sliding windows of 1,000 probes, and a z-test was performed on each window to determine whether the probes had a log_2 _ratio greater than zero. We were able to identify known amplification events in Dd2 surrounding *pfmdr1 *on chromosome 5 and *pfgch1 *on chromosome 12 [22] using a z-value cutoff of 18, which was used as a cutoff for the analysis below (Additional data file 2). Quantitative PCR had earlier determined that this isolate harbored 3-4 copies of *pfmdr1 *[29,30], and quantitative RT-PCR of our freshly cloned Dd2 line indicated the presence of three copies (data not shown). With our method, we did not detect a threefold increase in intensity for this region. Instead we saw a 1.6-fold intensity increase, with a log_2 _ratio of approximately 0.7 (Figure 1). Hybridization-based methods may not be linear with respect to copy number detection because of probe or scanner signal saturation. Linearity could possibly be achieved by shortening the hybridization times or by performing titrations. However, it would probably be more efficient to perform quantitative PCR than to collect whole-genome data. The goal of our study was to discover CNVs and not to extensively characterize them.

With a large number of probes to both coding and intergenic regions, we predicted that our method would allow us to delineate the breakpoints of amplification events with high accuracy, when comparing isolate genomic hybridizations with a reference isolate. To determine whether we could precisely detect the previously determined boundaries of amplification events, we scanned through the regions surrounding the amplifications and performed a paired *t*-test comparing the probe intensities on either side of each position on a chromosome with the window size dependent on the size of the amplification event. The position with the best *t*-statistic was considered to be the breakpoint of the amplification event, and reported ranges included positions with a *t*-statistic greater than 90% of the peak.

PCR analysis of the *pfmdr1 *amplification event in Dd2 previously estimated the breakpoints to be 888,335 on the 5' end and 970,240 on the 3' end [31]. Microarray-based mapping localized the amplification breakpoints at approximately 888,543 (888,393 to 888,689) and 970,202 (969,734 to 970,348) with a 2 kilobase (kb) window size (Figure 1). This amplification event could not be as precisely mapped because the breakpoints occur in intergenic tracts of monomeric A or T; nevertheless, we were still able to identify the region to within a few hundred bases. Our method also identified the GTP cyclohydrolase I amplification event in Dd2, which included three genes, namely PFL1145w, PFL1150c, and PFL1155w (*pfgch1*), confirming our initial report [22]. With our high-density microarray, we detected the breakpoints of this amplification as 971,195 (971,185 to 971,218) and 976,476 (976,448 to 976,525) using a 500 base pair window size. In HB3, the amplification event surrounding *pfgch1 *was much larger, about 161 kb, with breakpoints at 942,424 (942,144 to 942,717) and 1,103,325 (1,103,153 to 1,103,624) when scanned with a 4 kb window size. Our initial report indicated this amplification event contained 39 genes (PFL1125w to PFL1315w) [22], but our higher resolution microarray identified the breakpoints within the coding regions of PFL1125w and PFL1315w, suggesting a full-length CNV for 37 of the 39 genes (Additional data files 1 and 2).

### Polymorphism detection

**Figure 2:**
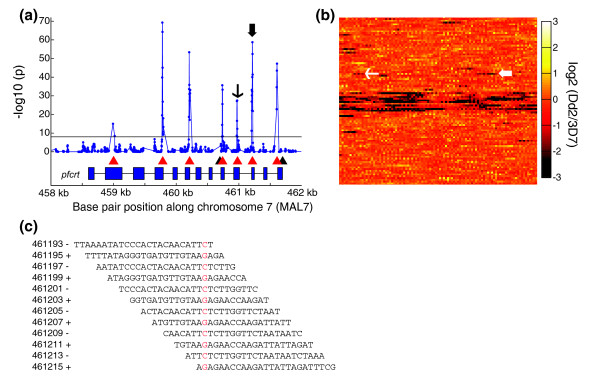
SNPs in Dd2. **(a) **Plot of -log_10 _*P *values for z-test (blue line) performed with Dd2 versus the 3D7 reference; detected are all of the reported single nucleotide polymorphisms (SNPs) in the *pfcrt *gene that were represented in the microarray (red triangles). Two SNPs were not detected (black triangles) because of the lack of unique probes to these positions. The thin and thick black arrows correspond to SNPs indicated in panel b. The *P*-value cutoff was 1 × 10^-8 ^(black line). **(b) **Visualization of probe intensity log ratios in Dd2 versus 3D7. Each probe is shown as a single pixel and is colored based on its log ratio. Continuous strips of dimmer pixels correspond to SNPs that map to multiple unique probes, as indicated by the thin and thick white arrows. **(c) **Probes were tiled through the genome with an offset of two to three base pairs of alternating strands. The position marked in red is the position of the SNP marked by the thick arrows in panels a and b. The base pair positions on the left indicate the start of the probe with respect to the plus strand of chromosome 5 and its orientation.

In addition to gene amplification events, drug resistance may also arise through the emergence of SNPs; thus, we next tested whether the microarray could globally detect these polymorphisms (Figure 2). As with CNV detection, probes that mapped to only one location in the 3D7 reference genome were used for the analysis. A z-test was performed on the difference in log intensities of the reference and test isolate hybridization with a sliding window of three overlapping probes. If there was no difference in the genomic sequence hybridizing to the probes, then the intensity differences formed a normal distribution centered on a mean of zero. The standard deviation of the normal distribution was empirically determined by replicate hybridizations of rank-invariant probes for the z-test. The appearance of SNPs between the experimental genomic DNA and the reference isolate resulted in intensities that were higher in the reference isolate. Based on our empirical data, we classified probes with a *P *value of less than 1 × 10^-8 ^and a higher mean in the reference hybridizations as ones containing polymorphisms in the isolate. Combining the data from the sliding windows of three probes enabled us to establish the boundaries within which the polymorphisms were contained. To predict the precise position of SNPs, we used an empirically determined model of loss of hybridization based on the SNP position in probes (Additional data file 3 [Figure S1]). An F-test was then performed with the model, based on the null hypothesis of the mean equaling zero, in order to position the polymorphism prediction at the peak of the *P *value.

Our SNP prediction validation was performed by comparing the 3D7 reference isolate to Dd2 and HB3, which have recently been sequenced to greater than 8× coverage by the Broad Institute [12]. In our analysis, we included high quality SNPs (Phred score >25 for each SNP and more than 25 base pairs from either end of an alignment or base pair call with Phred score <20) that were present in four or more reads (Dd2), and excluded SNPs in hypervariable subtelomeric regions defined in recent re-sequencing efforts [12]. Because the HB3 genome was assembled before SNP detection in the work by Volkman and coworkers [12], we were unable to examine individual HB3 sequence reads to determine coverage and quality values for SNPs and thus had a larger set of SNPs about which we were less confident. Initial analyses with the test set limited to SNPs that mapped to at least three unique probes on the microarray provided coverage of 75.9% for coding regions and 41.2% for noncoding regions, but resulted in suboptimal detection rates (Additional data file 3 [Table S1]). Therefore, the test set was limited to SNPs that mapped to six or more unique probes on the array, which restricted our microarray coverage to 58.5% of coding regions and 23.2% of noncoding regions. This produced a validation set of 1,737 SNPs in Dd2 and 3,344 SNPs in HB3 (Additional data file 3 [Table S2] and Additional data file 4).

From this set of high-quality SNPs we were able to identify 91.1% of SNPs (1,582 true positives, 155 false negatives) with a false discovery rate of 10.5% (185 false positives) when comparing two hybridizations of Dd2 with two reference hybridizations and using a *P *value cutoff of 1 × 10^-8^. Detection rate was defined as the percentage of the SNP validation set that was confirmed by microarray analysis; false discovery rate was the percentage of SNPs detected by microarray that were not polymorphic by Broad Institute sequencing [12]. For the HB3 SNPs, we detected 85.0% of SNPs (2,842 true positives and 502 false negatives) with a false discovery rate of 16.7% (569 false positives) using the same *P *value cutoff. The lower detection rate in HB3 may be due to the lack of sequence read information and thus less confidence in SNP identification from the original sequencing effort [12]. For Dd2 we also obtained similar lower detection rates when including lower-quality SNPs that had fewer than four reads. In *Saccharomyces cerevisiae*, Gresham and coworkers identified 96.2% of the published SNPs (944 out of 981) with a 95.9% false discovery rate (22,082 false positives) [25]. With a more stringent prediction signal cutoff, Gresham and coworkers [25] were able to obtain an impressively low false discovery rate of 0.87% with 81.7% detection using the well characterized model organism *S. cerevisiae*. In comparison, with a *P *value cutoff of 1 × 10^-5^, we detected 93.9% of SNPs in Dd2 with a 57.6% false discovery rate. Although we were unable to achieve false discovery rates of less than 1% because of the incomplete sequencing of the isolates and the nonclonal nature of parasite cultures, our SNP identification and false discovery rates approach those of conventional sequencing - the current gold standard (Tables 1 to 2 and Additional data file 3 [Figure S2]).

**Table 1:** SNP prediction results: Dd2 SNP call rates

z-test *P *value	True positives	False positives	Detection rate (%)	False discovery rate (%)
1 × 10^-5^	1,635	2,193	93.9	57.3
1 × 10^-8^	1,582	185	91.1	10.5
1 × 10^-10^	1,532	39	88.0	2.48

SNP, single nucleotide polymorphism.

**Table 2:** Previously published SNP prediction results: yeast SNP call rates

Prediction signal	True positives	False positives	Detection rate (%)	False discovery rate (%)
>0	944	22,082	96.2	95.9
>5	801	7	81.7	0.87
>5,347	760	0	77.5	0.00

Based on data from Gresham and coworkers [25]. SNP, single nucleotide polymorphism.

To scrutinize our false discovery and false negative rates, we used PCR amplification and subsequent sequencing of selected predicted polymorphisms with low *P *values classified as 'false positives', as well as 'false negative' polymorphisms with high probe coverage that were missed by our method. Any 'false positives' arising from indels present in sequencing reports were manually filtered and excluded from false discovery rates for Dd2 *P*-value cutoffs of 1 × 10^-8 ^or 1 × 10^-10^. Our results showed that 50% of the remaining high-quality 'false positives' were actually polymorphisms that were either different in the laboratory isolates that we analyzed or not detected by the re-sequencing project, similar to the 64% error rate described by Jiang and coworkers [26]. This suggests that our actual false discovery rate is approximately 6%. We did not detect 8.9% of the reported SNPs with good probe coverage in Dd2, and for all of these 'false negatives' sequenced there indeed was a polymorphism that was not detected by our arrays. More stringent *P*-value cutoffs increased the false negative rate but improved the false discovery rate. Additionally, the prediction of SNP location also increased with more stringent *P *values and higher density probe coverage. When the prediction signal contained more than ten probes that included the SNP, we were able to localize the position of the polymorphism prediction to within seven base pairs in more than 95% of cases and to within ten base pairs in more than 98% of cases. Even with weaker signals (four probes), we were able to localize approximately 95% of SNPs to within ten base pairs (Table 3).

**Table 3:** Accuracy of Dd2 SNP positioning

**Signal probes**	**0 bp (%)**	**± 2 bp (%)**	**± 5 bp (%)**	**± 10 bp (%)**
4 to 5	8.9	50.0	83.7	94.7
6 to 7	13.5	60.7	88.6	97.8
8 to 10	17.7	67.4	92.1	98.2
11 to 13	18.2	69.3	91.1	98.1

bp, base pairs; SNP, single nucleotide polymorphism.

### Comparison with previous studies

Previous microarray-based studies of *P. falciparum *have assessed genetic variability in the parasite [22,26,32] but these microarrays were not designed with the probe density and coverage to detect small changes in the genome that result from *in vitro *evolution or drug resistance. For example, the recent study by Jiang and coworkers [26] reports rates of polymorphic probes for genes across various laboratory isolates but fails to detect the hallmark mutation resulting in the K76T mutation in *pfcrt*, presumably because of insufficient probe coverage. The authors did not report individual polymorphisms identified in each of the laboratory isolates, and thus it was not possible to compare our methods directly. Another advantage of our current study is that the overlapping nature of probes allowed us to use one or two microarray replicate hybridizations to detect polymorphisms instead of three to four hybridizations required in previous studies [22,26].

### Detection of mutations in an engineered parasite line

**Figure 3:**
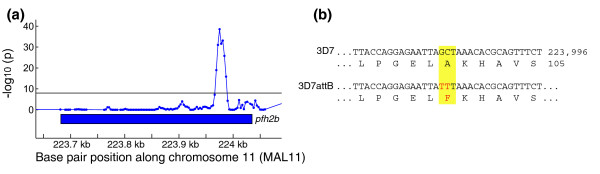
Mutation in 3D7^attB ^histone 2B. **(a) **Plot of -log *P*-value for z-test (blue line) performed with 3D7^attB ^versus the 3D7 reference detected a polymorphism in the histone 2B gene with a *P*-value cutoff of 1 × 10^-8 ^(black line). **(b) **Sequencing of the histone 2B gene in 3D7^attB ^revealed two consecutive point mutations resulting in a coding nonsynonymous mutation.

Classical genetic approaches are essential for elucidating gene function, but the transfection process and the subsequent drug selection are population bottlenecks. Emerging parasites may contain changes elsewhere in the genome that are undetected by standard controls but contribute to phenotype. To determine systematically additional mutations that arose or were selected in an engineered parasite line, we hybridized genomic DNA from 3D7^attB ^to the microarray. The 3D7^attB ^line was generated from 3D7 by the insertion of an attB recombination site for Bxb1 integrase flanked by the human *dhfr *gene as a selectable marker [33]. We detected a deletion of a subtelomeric region of chromosome 6 (1.38 to 1.41 megabases) and a polymorphism in the histone 2B gene (PF11_0062; Figure 3a and Additional data file 4). Sequencing revealed two novel point mutations resulting in a nonsynonymous mutation of A100F in this highly conserved gene (Figure 3b), strengthening the importance of whole genomic screening for undetected mutations in engineered lines.

### Selection and characterization of fosmidomycin resistance

Malarial parasites, unlike mammals, employ a nonmevalonate based 2-*C*-methyl-D-erythritol-4-phosphate (MEP) pathway for synthesis of essential isoprenoids such as ubiquinones and dolichols [34]. 1-Deoxy-D-xylulose 5-phosphate reductoisomerase (DXR), a key enzyme of the MEP pathway, catalyzes the conversion of 1-deoxy-D-xylulose 5-phosphate (DOXP) to MEP and is of particular therapeutic interest because of its requirement for isoprenoid synthesis in *P. falciparum *as well as several pathogenic eubacteria including *Bacillus anthracis*, *Helicobacter pylori*, *Yersinia pestis*, and *Mycobacterium tuberculosis *[35]. Recent clinical studies with the specific DXR inhibitor fosmidomycin have shown that this agent is well tolerated in humans and can cure *P. falciparum *infection, either alone or more potently in combination with clindamycin or artesunate [36-38]. These trials have yet to report resistance to fosmidomycin, including in areas with a high prevalence of parasite resistance to several other clinically used antimalarials.

**Figure 4:**
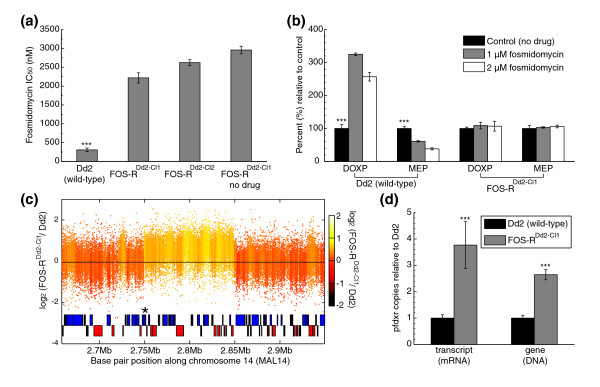
Fosmidomycin resistance. **(a) **Fosmidomycin 50% inhibitory concentration (IC_50_) values (means and standard deviations) were calculated from independent [^3^H]hypoxanthine assays in duplicate; the number of assays (*n*) is indicated in parenthesis. Drug susceptibility profiles of Dd2 (wild-type) (n = 7), FOS-R^Dd2-CL1 ^(n = 5), FOS-R^Dd2-CL2 ^(n = 3), and control FOS-R^Dd2-CL1^-no drug (n = 3). Tests for significant differences between the parental and drug-resistant isolates were performed using analysis of variance with Bonferroni posttests with α = 0.001; significant differences (*P *value < 0.001) are marked with three asterisks (***). **(b) **Effect of fosmidomycin on 1-deoxy-D-xylulose 5-phosphate (DOXP) and 2-*C*-methyl-D-erythritol-4-phosphate (MEP) biosynthesis in intact erythrocyte-free parasites exposed to fosmidoymicin for 24 hours and then labeled with [2-^14^C]pyruvic acid as a metabolic precursor. Means and standard deviations were calculated from four independent experiments performed in duplicate. Differences between control (no drug) Dd2 and fosmidomycin-treated Dd2 were significant by Student's *t*-test for DOXP (1 μmol/l drug: *P *value 1 × 10^-8^; 2 μmol/l drug: *P *value 1 × 10^-6^) and MEP (1 μmol/l drug: *P *value 4 × 10^-6^; 2 μmol/l drug: *P *value 5 × 10^-8^) and are marked with three asterisks (***). **(c) **To generate the plot, the log_2 _ratio of the intensity of each unique probe in FOS-R^Dd2-CL1 ^was divided by the intensity of each unique probe in Dd2. The probe log ratios were colored by the moving average over a 500 base pair window as indicated in the color bar. The amplification was approximately 100 kb and contained 23 genes including *pfdxr *(marked with an asterisk). **(d) **Quantitative RT-PCR analysis of *pfdxr *transcript level and quantitative PCR analysis gene copy number in FOS-R^Dd2-CL1 ^compared with Dd2. The means and standard deviations of two independent quantitative RT-PCR and quantitative PCR assays are represented. Significance differences (*P *value < 0.001) in ΔCt values were determined by Wilcoxon rank sum test and are marked with three asterisks (***).

To gain insight into how fosmidomycin resistance could evolve in *P. falciparum*, Dd2 parasites were exposed to drug *in vitro *and selected under stepwise increases in concentration. Two fosmidomycin-resistant clones, named FOS-R^Dd2-CL1 ^and FOS-R^Dd2-CL2^, were isolated from two separate flasks. These were then subjected to 72-hour [^3^H]hypoxanthine incorporation assays to determine the degree of resistance. Fosmidomycin 50% inhibitory concentration (IC_50_) values for FOS-R^Dd2-CL1 ^and FOS-R^Dd2-CL2 ^were 2,219 ± 136 and 2,623 ± 78 nmol/l, respectively, compared with an approximately eightfold lower IC_50 _value of 307 ± 49 nmol/l for the parental Dd2 line (Figure 4a). Fosmidomycin-resistant lines exhibited no change in their IC_50 _value for chloroquine compared with Dd2 (data not shown).

To confirm that fosmidomycin resistance was related to the MEP pathway, infected erythrocytes were pretreated for 24 hours with fosmidomycin and the parasites released by saponin treatment. Intact erythrocyte-free parasites were then metabolically labeled with [2-^14^C]pyruvic acid in the presence of fosmidomycin. As expected, the Dd2 parental line showed DOXP accumulation while MEP synthesis was reduced in a dose-dependent way in treated parasites (Figure 4b). In contrast, the synthesis of DOXP and MEP was unaffected by drug in fosmidomycin-resistant parasites (Figure 4b). The fact that DOXP did not accumulate in a dose-dependent manner in Dd2 could be due to negative feedback regulation, as previously observed in *P. falciparum *[39,40]. As a control, the synthesis of adenosine metabolites through the purine pathway was analyzed under the same treatment conditions using [2-^3^H]adenosine as the metabolic precursor (Additional data file 3 [Figure S3AB]). No significant differences in [2-^3^H]adenosine uptake were observed between untreated and fosmidomycin-treated parasites both in Dd2 and FOS-R^Dd2-CL1^. Similarly, no significant differences in parasite growth were observed during the 24-hour treatment (Additional data file 3 [Figure S3C]). To test the stability of the fosmidomycin-resistant phenotype, frozen stocks of the FOS-R^Dd2-CL1 ^clone were thawed and parasites maintained in the absence of drug pressure for 6 weeks. Fosmidomycin assays with this parasite line yielded an IC_50 _value of 2,958 ± 101 nmol/l, which was comparable with *in vitro *selected resistant lines maintained under drug (Figure 4a). These studies demonstrate that fosmidomycin resistance can be readily and stably acquired *in vitro *by selective pressure on culture-adapted malarial parasites.

To investigate the genetic basis of this drug resistance, DNA from these fosmidomycin-resistant mutants and the parental Dd2 line were hybridized to the arrays. Unexpectedly, we identified a single large amplification event in the genome, on chromosome 14 of the fosmidomycin-resistant clones compared with Dd2. This CNV contained 23 genes (PF14_0641 to PF14_0663) and was approximately 100 kb in size (Figure 4b and Additional data file 2). The first gene in the amplification event was identified as *pfdxr*, the putative target of fosmidomycin in *Plasmodium*, and the amplification event included the entire upstream intergenic region for this gene. The log_2 _ratio of FOS-R^Dd2-CL1^/Dd2 intensity for the region was approximately 0.7, suggesting the presence of three copies of this region. Quantitative RT-PCR demonstrated an approximately 3.8-fold increase in the *pfdxr *transcript level and a approximately 2.7-fold increase in gene copy number in FOS-R^Dd2-CL1 ^when compared with Dd2 (Figure 4d). These investigations suggest that fosmidomycin pressure selected for parasites that had amplified the *pfdxr *gene. Finally, the entire open reading frames of *pfdxr *and *pfdxs *(DOXP synthase, MAL13P1.186), the most likely candidate resistant determinants in the mevalonate-independent isoprenoid synthesis pathway, were sequenced and analyzed for the presence of mutations. No sequence changes were observed in comparison with the wild-type parasite line.

### Development of software tools

We created a powerful MATLAB-based graphical user interface that implements our methods to visualize SNPs, perform SNP prediction, and visualize gene CNVs, allowing researchers to interpret the microarray data. This software is freely available as a standalone application without restrictions for Mac OS X and Windows platforms from our website [41].

## Discussion

Recent studies suggest that CNVs of genes, involved in diverse processes from cell cycle regulation to sexual differentiation, may be a common strategy used by *P. falciparum *to overcome environmental stresses and drug pressure [22,31,42]. The amplification of GTP cyclohydrolase I, the first enzyme of the folate biosynthesis pathway, is hypothesized to shuttle more substrate into this pathway in the presence of various antifolate antimalarials and was missed in the sequencing efforts [22]. This is not surprising because sequencing the human genome missed a large number of CNVs subsequently described by various microarray analyses [43-46]. Compared with the microarrays used in all of these studies, our method not only allows for more powerful statistics with hundreds to thousands of unique probes for most genes, but it also allows for accurate break point mapping with probes to intergenic regions. We applied our method to fosmidomycin-resistant parasites and identified an increase in gene copy number of the putative target *pfdxr *as a plausible mechanism for *in vitro *derived resistance. This approach can be extended to additional antimalarial compounds to determine candidate mechanisms of resistance and potentially identify drug targets when the mechanism of action is unknown.

Although CNVs in *pfmdr1 *and *pfgch1 *have been reported, the amplification of *pfdxr *is the first example of resistance due to the amplification of the target enzyme of a drug in *Plasmodium*. These amplification events may be common methods for parasites to increase transcription of important genes in light of recent studies confirming the inflexibility of the programmed asexual stage transcriptome to respond to drug pressure [47] because parasites have evolved in a relatively stable environment of the human host. Elucidating the mechanism of resistance to fosmidomycin is important in view of its promising role in clinical combination therapies [36-38]. Although the volume of blood obtained with a finger stick may not yield enough parasite DNA to do these experiments, 5 to 10 ml of leukocyte-depleted infected blood may yield enough material for a successful hybridization. In additional, clonal field isolates may be pushed into short-term culture for characterization by microarray.

Our method allows for rapid and accurate detection of SNPs in the parasite, as illustrated by our detection of known SNPs in the established drug resistance genes *dhfr-ts *and *pfcrt *in Dd2 (Figure 2a, b). This method achieved greater than 90% SNP detection with low false positive rates of about 10%, similar to those reported by re-sequencing efforts [12]. Polymorphisms identified by microarray improve the confidence of SNPs identified by sequencing, and perhaps these two methods can be implemented in a complementary way for large-scale detection of genetic variation. The existence of up to 13 probes covering a single base pair location provided the equivalent of multiple-fold coverage that sequencing can provide. Insertions and deletions (indels) are common mutations that we also wished to evaluate by microarray. Unfortunately, indels were not systematically reported by re-sequencing efforts, and thus we were unable to determine the accuracy of indel identification by microarrays compared with sequencing. However, we did find that many 'false positives' were actually insertions or deletions present in the sequencing reports, suggesting that our method can also accurately detect indels. We also identified nonsynonymous point mutations in the engineered parasite line 3D7^attB^, illustrating the utility of our microarray for discovering undetected and unexpected genetic changes that may arise or be selected during genetic manipulation and subsequent selection of parasites.

Traditional sequencing methods, like those used to sequence all *P. falciparum *isolates to date, are considered to be the gold standard. Yet their consensus accuracies of 99.99% would still result in thousands of potential errors. Furthermore, shotgun sequencing methods involve transforming *Escherichia coli *with parasite DNA, which can be difficult with *P. falciparum *because of its extremely high AT content. PCR amplification steps may also bias the results, and traditional sequencing methods have missed CNVs that are an important mechanism for drug resistance in *P. falciparum *and other species, including humans [43-45]. Next generation sequencing technologies are quickly becoming more affordable and are beginning to be used to assess genetic variability in a number of organisms with SNP detection rates that are comparable [48-50] to those of our microarray-based method. However, these methods also have their caveats. With a highly repetitive AT-rich genome like *P. falciparum*, masking or filtering steps may limit the detection of polymorphisms in many intergenic regions and coding regions. Our hybridization-based method achieves detection rates similar to those of sequencing and is much faster, requiring a single overnight experiment followed by rapid computational analysis with results presented in a form readily accessible to researchers.

## Conclusions

Our whole-genome approach will prove particularly helpful when elucidating mechanisms of drug resistance *in vitro *or in clinical isolates of *P. falciparum*, as demonstrated by the identification of *pfdxr *amplification in fosmidomycin resistance. This method can also be applied to screening populations of *P. falciparum *and tracking the spread of drug resistance, to discover the genetic basis of other phenotypic changes, and to address more fundamental questions in *P. falciparum *biology, such as the basal rates of point mutations and CNVs in laboratory culture. By probing genetic variability in *P. falciparum *with a single hybridization in a single overnight experiment, this tiled microarray methodology will allow researchers using the accompanying software to quickly and accurately ascribe phenotypic variations to changes at the gene level. This will facilitate a deeper understanding of how the parasite is evolving worldwide both in field and laboratory-adapted field isolates, supporting the new drive to reduce the burden of malaria more effectively.

## Materials and methods

### DNA methods

Cultured isolates of 3D7 (MRA-151), Dd2 (MRA-156), HB3 (MRA-155), and 3D7^attB ^(MRA-845) were obtained from the Malaria Research and Reference Reagent Resource Center (MR4; American Type Culture Collection, Manassas, VA, USA). *P. falciparum *parasite lines were propagated in human erythrocytes as previously described [33,51]; Dd2 was freshly cloned from the MR4 isolate. Genomic DNA was isolated by standard phenol-chloroform extraction. Fifteen micrograms of genomic DNA from each isolate and 10 ng each of Bio B, Bio C, Bio D, and Cre Affymetrix control plasmids (Affymetrix Inc., Santa Clara, CA, USA) were fragmented with DN*ase*I and end-labeled with biotin [52]. The samples were hybridized to the microarrays overnight at 45°C in Affymetrix buffers, washed, and scanned [22].

### Background subtraction and microarray normalization

Background subtraction was performed for each array using Affymetrix eukaryotic background control probes. Because of the varying background hybridization of probes based on their GC content, the background probes were split into bins based on GC content, and the mean for each bin was calculated. The appropriate background value, based on GC content, was subtracted from probes used for the analyses. As described above, the arrays were normalized to a synthetic baseline array constructed by taking the means across all probes for all arrays used in the experiment. The probe intensities were log transformed and placed into 100 equally sized and spaced bins. After removing outliers, the median for each bin was set to the median of the corresponding bin in the synthetic baseline microarray.

### Probe selection

The microarray contains probes to the *P. falciparum *3D7 nuclear, mitochondrial, and apicoplast genomes, as well as isolate-specific probes for HB3 and Dd2, *P. knowlesi *specific probes, and eukaryotic control probes (standard Affymetrix and *Arabidopsis thaliana*). Unique probe mapping was determined by blasting *P. falciparum *probe sequences against the reference genome (PlasmoDB version 5.3) and determining the number of perfect matches. We excluded from our analysis any probes that had more than one perfect match.

### Nucleotide sequencing

Primers were designed to amplify 400 to 500 base pairs of Dd2 genomic DNA with the false positive or false negative position near the center. Independent PCR products were sequenced by ABI sequencing (Applied Biosystems Inc., Foster City, CA, USA).

### *In vitro *selection of fosmidomycin-resistant parasites and antimalarial drug assays

The *P. falciparum *Dd2 line (a mefloquine-resistant derivative of W2 originally from Indochina) was used for the selection of fosmidomycin resistance. For the drug selection experiment, approximately 2 × 10^10 ^mixed stage parasites were exposed to 100 nmol/l of fosmidomycin for 5 days. Cultures were maintained carefully by smearing every day and feeding twice daily with drug-containing RPMI media. After initial drug treatment, fosmidomycin drug concentration was increased to 400 nmol/l and kept at this level for the following 7 days. At this drug concentration, dying asexual-stage parasites were observed by microscopic examination of Giemsa-stained smears. The fosmidomycin drug selection level was then increased to 700 nmol/l, which eliminated asexual stage parasites from detection by microscopy, and cultures were further maintained at this drug level throughout the experiment. Reappearance of healthy asexual stage parasites growing in the presence of drug were observed approximately 6 weeks after the start of the selection experiment. Once parasitemia reached 2% to 3%, frozen stocks of fosmidomycin-resistant parasites were prepared using Glycerolyte 57 (Baxter Healthcare, Deerfield, IL, USA). During the entire selection process 30% to 40% red blood cells were replaced with freshly washed cells once a week. Drug selected parasites were cloned by limiting dilution in 96-well tissue culture plates in the presence of 700 nmol/l of fosmidomycin, with an inoculum of 0.5 infected red blood cells per well. Parasite clones were detected after 3 weeks of growth using the *P. falciparum *lactate dehydrogenase-specific Malstat assay [53].

Parasites were phenotypically characterized for their drug susceptibility profiles using [^3^H]hypoxanthine incorporation assays, as described previously [54]. The response to fosmidomycin and chloroquine was measured *in vitro *in 96-well plates using 72-hour [^3^H]hypoxanthine assays, starting with an initial parasitemia of 0.4% to 0.5%. IC_50 _values were calculated using linear regression [54,55].

### Metabolite profiling

One cycle after sorbitol synchronization, predominantly ring-stage cultures at about 10% parasitemia were treated for 24 hours with 1 μmol/l or 2 μmol/l fosmidomycin. Untreated cultures were incubated in parallel. After treatment, parasites were isolated from their host erythrocytes by incubating with 20 pellet volumes of 0.015% saponin in phosphate-buffered saline (PBS), and then washed three times with PBS at room temperature. Parasitemia and parasite morphology were determined by microscopic analysis of Giemsa-stained blood smears immediately before and after saponin treatment. Untreated or treated intact erythrocyte-free parasites were resuspended in RPMI 1640 medium and labeled for 1 hour with 14 μmol/l [2-^14^C]pyruvic acid (10 to 40 mCi/mmol; Perkin Elmer, Waltham, MA, USA) or 1 μmol/l [2-^3^H]adenosine (23 Ci/mmol; Amersham Biosciences, Piscataway, NJ, USA) in the absence (controls) or presence of fosmidomycin. After incubation, parasites were centrifuged and washed twice with ice-cold PBS. Parasites were immediately extracted with ethanol/water (1:1 vol/vol; 1 × 0.3 ml at 55°C for 1.5 hours) [39] for subsequent high-performance liquid chromatography (HPLC) analysis of DOXP and MEP metabolites. Purine metabolites were extracted by perchloric acid treatment. Briefly, samples were mixed 1:6 (vol/vol) with 0.5 mol/l HClO_4_, vigorously mixed, and incubated for 20 minutes at 4°C. Samples were centrifuged and supernatants were neutralized with 5 mol/l KOH for 20 minutes at 4°C. All extracts were filtered through YM-10 Centricon columns (MW retention = 10000; Amicon, Millipore, Billerica, MA, USA). Analyses of metabolites were accomplished by using 2 × 10^8 ^parasites.

At the beginning of treatment, 1 ml culture from each condition was used in [^3^H]hypoxanthine incorporation assays to test growth inhibition. After 12 hours of treatment, [^3^H]hypoxanthine was added at a final concentration of 5 μCi/ml. After an additional 12 hours of incubation, cells were harvested according to the method described by Desjardins and colleagues [54].

DOXP and MEP intermediates were analyzed by HPLC as described previously [39]. Briefly, the ethanol/water fractions were analyzed using a reverse-phase column (Luna C_18_[2], 150 × 4.6 mm, 3 μm; Phenomenex, Torrance, CA, USA). The eluants were 20 mmol/l *N, N*-dimethylhexylamine in 10% methanol with the pH adjusted to 7.0 with formic acid (solution A) and 50% methanol containing 2 mmol/l *N, N*-dimethylhexylamine, pH 7.0 (solution B). The HPLC gradient was 10% to 50% methanol in 50 minutes. The eluant was monitored at 270 nm at a flow rate of 0.75 ml/minute. The adenosine metabolites inosine, hypoxanthine and inosine monophosphate were analyzed in a reverse-phase (Luna C_18_[2], 150 × 4.6 mm, 3 μm; Phenomenex, Torrance, CA, USA) ion-pair HPLC system. The mobile phases were 8 mmol/l tetrabutylammonium bisulfate (Fluka, Sigma Aldrich, St. Louis, MO, USA) and 100 mmol/l KH_2_PO_4 _with the pH adjusted to 6.0 with KOH (solution A), and 30% acetonitrile containing 8 mmol/l tetrabutylammonium bisulfate and 100 mmol/l KH_2_PO_4 _(pH 6) as solution B. The HPLC gradient was from 0% to 100% solution B in 20 minutes. The eluant was monitored at 254 nm and the flow rate was 1 ml/minute. Aliquots from both HPLC systems were collected based on UV detection of internal standards and subjected to liquid scintillation counting. For the comparison of the influence of fosmidomycin treatment on the biosynthesis of the different metabolites, the same numbers of treated or untreated parasites were analyzed.

### Quantification of *pfdxr *copy number and transcript levels

Real-time PCR and real-time RT-PCR methods were employed to quantify *pfdxr *gene copy number and transcript levels respectively in fosmidomycin-resistant and parental lines. Genomic DNA extractions were performed using Qiagen DNeasy kits (Qiagen, Hilden, Germany), and RNA samples were prepared from the trophozoite-stage synchronized parasites using Trizol (Invitrogen, Carlsbad, CA, USA). DNase-treated RNA was reverse transcribed using the SuperScript^® ^III First-Strand Synthesis System (Invitrogen, Carlsbad, CA, USA). Genomic DNA and cDNA templates (at concentrations 1, 0.5, 0.25, 0.125, 0.625, 0.0312, and 0.01562 ng) were PCR amplified using the QuantiTect SYBR Green PCR Kit (Qiagen, Hilden, Germany) with *pfdxr*-specific primers (forward: 5'-TCAAGAACTTGCGATATTATAGAGG; reverse: 5'-TTGGCTCAGGTTTCAACTCTTACAT) or actin-specific primers (forward: 5'-AGCAGCAGGAATCCACACA; reverse: 5'-TGATGGTGCAAGGGTTGTAA). The 2^-ΔΔCt ^method was employed to assess the copy number and transcript level (Applied Biosystems Inc., Foster City, CA, USA).

### Database version

All gene information and base pair positions were taken from PlasmoDB version 5.3 [56].

### Microarrays and probe definition files

The microarray probe definition file, raw microarray data (CEL files), and result files from our analysis are available for download from our website [41]. Microarrays can only be custom ordered in bulk quantities, and thus upon request, the authors will coordinate groups wishing to obtain microarrays and have authorized Affymetrix to sell the microarrays to anyone who wishes to buy them.

## Abbreviations

CNV: copy number variation; DOXP: 1-deoxy-D-xylulose 5-phosphate; DXR: 1-deoxy-D-xylulose 5-phosphate reductoisomerase; HPLC: high-performance liquid chromatography; IC_50_: 50% inhibitory concentration; kb: kilobase; MEP: 2-*C*-methyl-D-erythritol-4-phosphate; MOID: match-only integral distribution; MR4: Malaria Research and Reference Reagent Resource Center; PBS: phosphate-buffered saline; RT-PCR: reverse transcription polymerase chain reaction; SNP: single nucleotide polymorphism.

## Authors' contributions

NVD, ABSS, MBC, DAF, and EAW conceived and designed the experiments. NVD, ABSS, MBC, SJW, SERB, RTE, and DP performed the experiments. SB and EAW designed the microarray. NVD developed analysis methods and analyzed the microarray data. SJW, DJP, SKV, DFW, and YZ contributed materials, data, and ideas. NVD, ABSS, MBC, DAF, and EAW wrote the paper. SJW, SERB, RTE, DJP, and SKV revised drafts of the paper.

## Additional data files

The following additional data are included with the online version of this article: a table listing MOID gene present calls and average log_2 _ratios for Dd2, HB3, 3D7^attB^, and FOS-R^Dd2-CL1 ^(Additional file 1); a table listing CNVs predicted by our algorithm in Dd2, HB3, 3D7^attB^, and FOS-R^Dd2-CL1 ^(Additional data file 2); supplementary tables S1 and S2, and figures S1 to S3 (Additional data file 3); and a table listing polymorphisms predicted by our algorithm in Dd2, HB3, 3D7^attB^, and FOR-R^Dd2-CL1 ^(Additional data file 4).

## Supplementary Material

Additional data file 1 Presented is a table listing MOID gene present calls and average log_2 _ratios for Dd2, HB3, 3D7^attB ^and FOS-R^Dd2-CL1^ Click here for file

Additional data file 2 Presented is a table listing CNVs predicted by our algorithm in Dd2, HB3, 3D7^attB ^and FOS-R^Dd2-CL1^. Click here for file

Additional data file 3 Table S1 contains SNP prediction results for Dd2. Table S2 contains SNP test set filtering information. Figure S1 is the probe behavior based on SNP position. Figure S2 is a genome-wide view of polymorphisms detected by microarray and sequencing in Dd2 relative to 3D7. Figure S3 contains control experiments for fosmidomycin resistance metabolite profiling. Click here for file

Additional data file 4 Presented is a table listing polymorphisms predicted by our algorithm in Dd2, HB3, 3D7^attB^, and FOR-R^Dd2-Cl1^. Click here for file
