# Supplementary material for: Use of high-density tiling microarrays to identify mutations globally and elucidate mechanisms of drug resistance in Plasmodium falciparum
Source: Genome Biol. 2009 Feb 13;10(2):R21. doi: 10.1186/gb-2009-10-2-r21 (PMC2688282; doi:10.1186/gb-2009-10-2-r21)
Supplement: Additional data file 3 — Table S1 contains SNP prediction results for Dd2. Table S2 contains SNP test set filtering information. Figure S1 is the probe behavior based on SNP position. Figure S2 is a genome-wide view of polymorphisms detected by microarray and sequencing in Dd2 relative to 3D7. Figure S3 contains control experiments for fosmidomycin resistance metabolite profiling. [file gb-2009-10-2-r21-S3.doc]

**Table S1** SNP prediction results for Dd2. When evaluating SNPs that mapped to at least three unique probes on our microarray, we achieved suboptimal results. However we did still detect polymorphisms for genome positions that had coverage by less than six unique probes, albeit with less confidence. The false positives reported here were not manually filtered to remove those caused by indels, and thus the false discovery rate may be overestimated.

|  | **Dd2 SNP Call Rates** | | | |  |
| --- | --- | --- | --- | --- | --- |
| **z-test p-value** | **True Positives** | **False Positives** | **Detection Rate (%)** | **False Discovery Rate (%)** | |
| 1×10^-5^ | 1951 | 2784 | 89.3 | 58.8 | |
| 1×10^-8^ | 1851 | 291 | 84.7 | 13.6 | |
| 1×10^-10^ | 1768 | 78 | 80.9 | 4.23 | |

**Table S2** SNP test set filtering. The most stringent step of filtering out SNPs for this analysis was the first step where we asked that SNPs not be within 25 base pairs of the end of an alignment as produced by the original sequencing efforts[12], or not be within 25 base pairs of a base pair call with Phred<20, or not be located in nonsubtelomeric regions. Further restrictions were that SNPs had to be present in 4 or more reads for Dd2. The last two columns are restrictions imposed by probes on the microarray.

|  | **SNP Test Set Filtering** | | | | |  |
| --- | --- | --- | --- | --- | --- | --- |
| **Isolate** | **Column A:**  **Total SNPs  by Broad sequencing** | **Column B:**  **SNPs not within 25 bp of unknown, less confident, or subtelomeric sequence** | **Column C:**  **SNPs in Column B with ≥4 reads (Dd2) and no mixed reads (Dd2 & HB3)** | **Column D:**  **SNPs in Column C with coverage of ≥3 unique probes** | **Column E:**  **SNPs in Column C with coverage of ≥6 unique probes** | |
| Dd2 | 17546 | 4790 | 3051 | 2185 | 1737 | |
| HB3 | 30753 | 7097 | 7045 | 4436 | 3344 | |


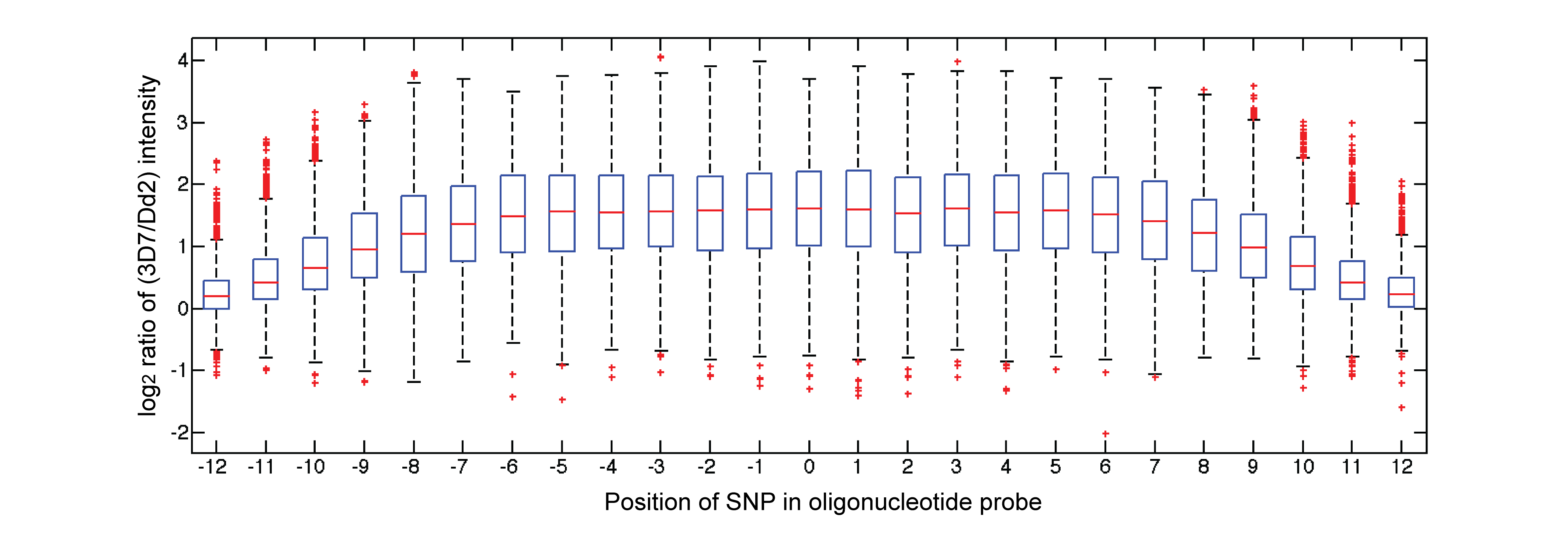


**Figure S1** Empirically based model of probe behavior based on SNP position. The empirical results for SNP position-based changes in log_2_ ratio of intensity for Dd2 correlated well with previous results in yeast[25] and were used to create the model. Position 0 indicated the center of the oligonucleotide probe, and the signal decreased when the SNP was more than 6 base pairs from the center of the probe. Model:


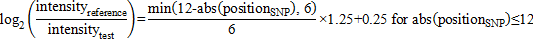


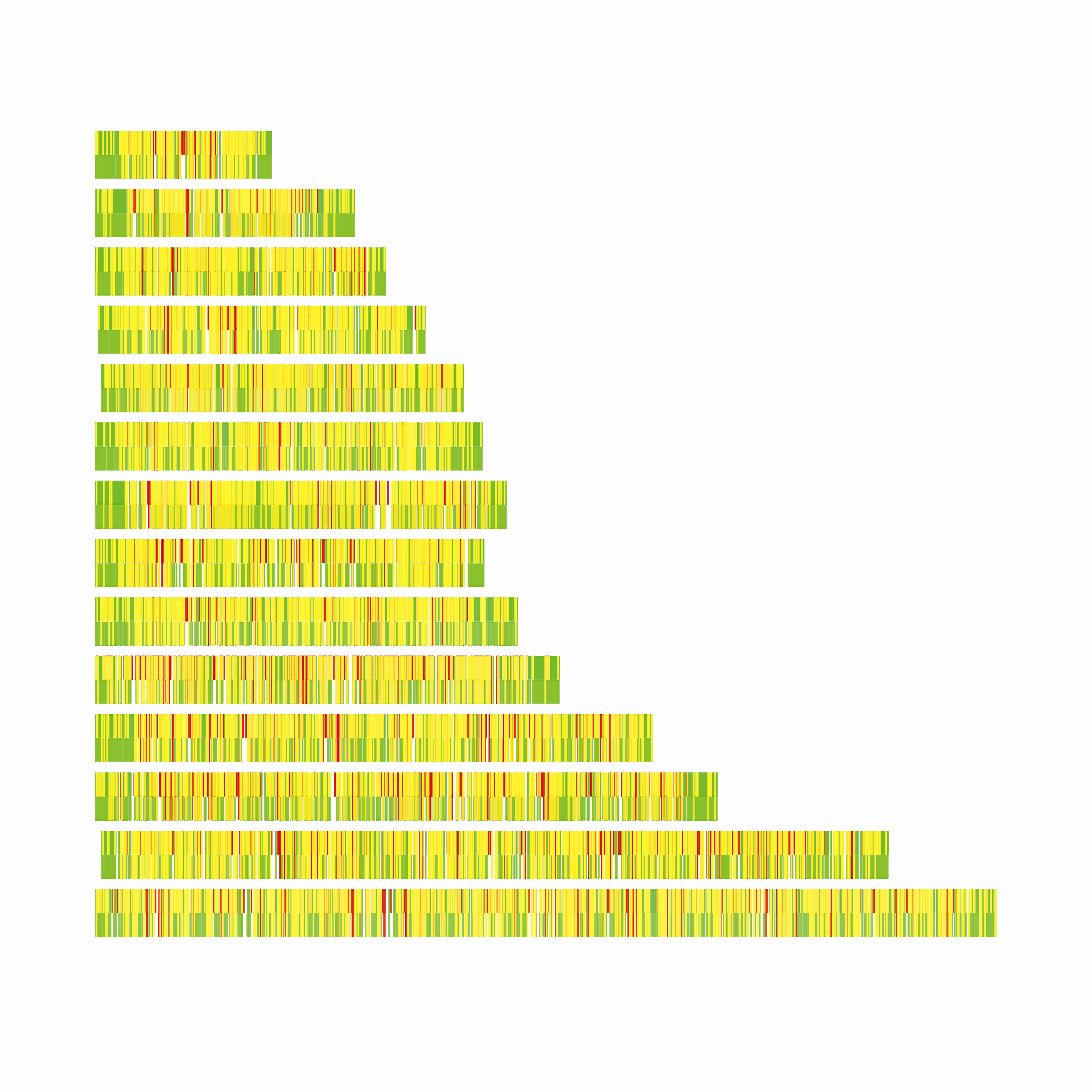
MAL1

MAL2

MAL3

MAL4

MAL4

MAL5

MAL6

MAL7

MAL8

MAL9

MAL10

MAL11

MAL12

MAL13

MAL14

**Figure S2** Genome-wide view of polymorphisms detected by microarray and sequencing in Dd2 relative to 3D7. Along each chromosome, polymorphisms identified by microarray are colored green. All SNPs identified by sequencing are colored red along the top half of each chromosome, and SNPs with only >4 reads and no mixed reads are colored red along the bottom half of each chromosome. Polymorphisms identified by both methods are yellow.

**
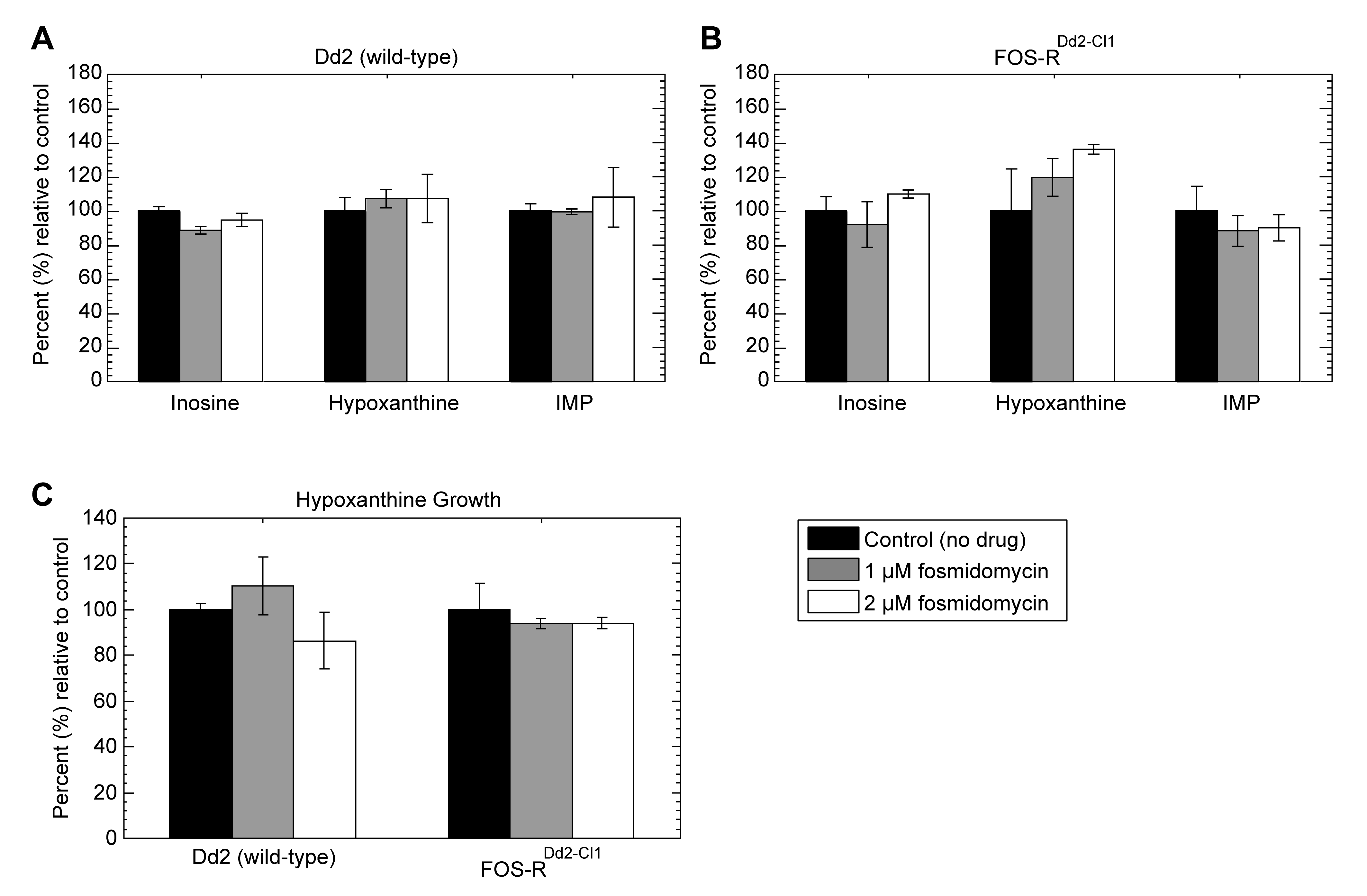
**

**Figure S3** Control experiments for fosmidomycin resistance metabolite profiling. **A** and **B** Effect of fosmidomycin on purine metabolite biosynthesis in intact erythrocyte-free parasites (**A** = Dd2 and **B** = FOS-R^Dd2Cl1^) labeled with [2-^3^H]adenosine as the metabolic precursor. **C** Parasite growth was measured by [^3^H]-hypoxanthine incorporation. The average and standard deviation of four independent experiments in duplicate are represented. No significant differences were noted. Significance was determined by Student’s t-test (p-value<0.01) with at least 30% inhibition outside the error range.
